# Aortic stenosis and mitral regurgitation modify the effect of venoarterial extracorporeal membrane oxygenation on left ventricular function in cardiogenic shock

**DOI:** 10.1038/s41598-022-21501-z

**Published:** 2022-10-12

**Authors:** Petr Ostadal, Dagmar Vondrakova, Michaela Popkova, Matej Hrachovina, Andreas Kruger, Marek Janotka, Jan Naar, Otomar Kittnar, Petr Neuzil, Mikulas Mlcek

**Affiliations:** 1 Cardiovascular Center, Na Homolce Hospital, Roentgenova 2, 15000 Prague, Czech Republic; 2 Department of Physiology, First Faculty of Medicine, Charles University in Prague, Prague, Czech Republic

**Keywords:** Blood flow, Cardiac device therapy

## Abstract

Venoarterial extracorporeal membrane oxygenation (VA-ECMO) is widely used in the treatment of patients experiencing cardiogenic shock (CS). However, increased VA-ECMO blood flow (EBF) may significantly impair left ventricular (LV) performance. The objective of the present study was to assess the effect of VA-ECMO on LV function in acute CS with concomitant severe aortic stenosis (AS) or mitral regurgitation (MR) in a porcine model. Eight female swine (45 kg) underwent VA-ECMO implantation under general anaesthesia and mechanical ventilation. Acute CS was induced by global myocardial hypoxia. Subsequently, severe AS was simulated by obstruction of the aortic valve, while severe MR was induced by mechanical destruction of the mitral valve. Haemodynamic and LV performance variables were measured at different rates of EBF rates (ranging from 1 to 4 L/min), using arterial and venous catheters, a pulmonary artery catheter, and LV pressure–volume catheter. Data are expressed as median (interquartile range). Myocardial hypoxia resulted in declines in cardiac output to 2.7 (1.9–3.1) L/min and LV ejection fraction to 15.2% (10.5–19.3%). In severe AS, increasing EBF from 1 to 4 L/min was associated with a significant elevation in mean arterial pressure (MAP), from 33.5 (24.2–34.9) to 56.0 (51.9–73.3) mmHg (P ˂ 0.01). However, LV volumes (end-diastolic, end-systolic, stroke) remained unchanged, and LV end-diastolic pressure (LVEDP) significantly decreased from 24.9 (21.2–40.0) to 19.1 (15.2–29.0) mmHg (P ˂ 0.01). In severe MR, increasing EBF resulted in a significant elevation in MAP from 49.0 (28.0–53.4) to 72.5 (51.4–77.1) mmHg (P ˂ 0.01); LV volumes remained stable and LVEDP increased from 17.1 (13.7–19.1) to 20.8 (16.3–25.6) mmHg (P ˂ 0.01). Results of this study indicate that the presence of valvular heart disease may alleviate negative effect of VA-ECMO on LV performance in CS. Severe AS fully protected against LV overload, and partial protection was also detected with severe MR, although at the cost of increased LVEDP and, thus, higher risk for pulmonary oedema.

## Introduction

Extracorporeal membrane oxygenation in the venoarterial configuration (VA-ECMO) is an established method that offers circulatory support in the most severe conditions of circulatory failure, including cardiogenic shock (CS). However, evidence supporting the use of VA-ECMO in CS is insufficient, with promising results only from retrospective or small randomized studies^1,2^; nevertheless, large multicenter trials are ongoing^3–5^.

Insertion of VA-ECMO in case of circulatory collapse enables rapid restoration and maintenance of adequate tissue perfusion; however, it may be associated with unfavorable consequences for the failing myocardium because the outflow part increases not only arterial blood pressure but also left ventricular (LV) afterload^6^. The increased afterload may cause LV overload and further reduction of LV performance, especially if the LV systolic function is already severely compromised. The unfavorable effect of increased extracorporeal blood flow (EBF) on LV performance during ECMO therapy has been described in several experimental and clinical studies^7–15^. Progressive LV overload is associated with increased left atrial pressure and frequently with subsequent severe pulmonary oedema, resulting in a critical condition that often requires urgent intervention (e.g., LV unloading)^16,17^.

In the presence of severe valvular heart disease VA-ECMO may, however, influence LV function in a different manner^18^. In particular, aortic stenosis (AS) or mitral regurgitation (MR) are not infrequent causes or modulators of severe CS requiring mechanical circulatory support^18–20^. The effect of VA-ECMO on LV function in CS with AS or MR has not been described. The aim of our study was, therefore, to assess LV functional variables at different VA-ECMO blood flow levels in a porcine model of acute CS with severe AS or MR.

## Methods

### Animal model

Eight female swine (*Sus scrofa domestica*, Large White × Landrace crossbreed), four to five months of age, and a mean body weight of 45 kg were used. Full details of the animal model used in the present study has been reported earlier^21^.

Briefly, after a 24 h fasting, general anaesthesia was induced by administration of midazolam (0.3 mg/kg intramuscular) and ketamine hydrochloride (15–20 mg/kg intramuscular). Initial propofol and morphine boluses (2 mg/kg intravenous [IV] and 0.1–0.2 mg/kg IV, respectively) were administered, followed by orotracheal intubation. Continuous IV infusions of propofol (6–10 mg/kg/h) and morphine (0.1–0.2 mg/kg/h) were used to maintain anaesthesia. The depth was adjusted according to physiological parameters, pupillary photoreactions, corneal and palpebral reflexes, lacrimation, and spontaneous movements^21^.

Potassium chloride (2 mEq/kg), in conjunction with general anaesthesia overdose, was used to euthanize the animals at the conclusion of the experiment.

Bilateral femoral (arterial and venous), carotid and jugular approaches were used for multiple sheath insertions using a standard percutaneous Seldinger technique. An initial rapid IV infusion of 1000 mL normal saline was administered after anaesthesia induction to correct hypovolemia caused by 24 h of fasting, followed by continuous IV drip at a rate of 100–500 mL/h to reach and maintain a mean right atrial pressure of 5–7 mmHg. An unfractionated heparin IV bolus (100 U/kg) was administered after vascular sheaths placement, followed by continuous IV infusion of 50 U/kg/h to maintain activated clotting time of 200–250 s. Values were monitored every hour using a microcoagulation system (Hemochron Jr Signature Plus Microcoagulation System, ITC, Piscataway, NJ, USA)^21^.

Ventilation was provided using a ventilator (Hamilton G5, Hamilton Medical AG, Switzerland) set to the INTELLiVENT–Adaptive Support Ventilation mode. The ventilator was set to maintain an oxygen saturation (SpO_2_) of 95–99% and an end-tidal carbon dioxide (CO_2_) pressure of 4.8–5.6 kPa^21^.

### VA-ECMO

The VA-ECMO setting used in our study has already been described elsewhere^7,21^. The ECMO circuit consisted of a console (Cardiohelp, Getinge, Germany), centrifugal blood pump and tubing set, a membrane oxygenator (Getinge, Germany), and a mechanical gas blender (Sechrist Industries, Inc, Anaheim, CA, USA). A venous cannula (21 Fr, Getinge, Germany) and arterial cannula (15 Fr, Getinge, Germany) were inserted percutaneously using the standard Seldinger technique into the femoral vein and artery. The venous inflow cannula was inserted into the right atrium (the tip position was verified using fluoroscopy), and the femoral arterial outflow cannula was inserted into the femoral artery with the tip placed in the descending aorta. Blood gas values leaving the oxygenator were continuously monitored (CDI™ Blood Parameter Monitoring System 500, Terumo Cardiovascular Systems Corporation, Elkton, MD, USA). The oxygen/air flow was repeatedly adjusted to maintain a partial pressure of oxygen (*Pa*O_2_) and partial pressure of CO_2_ (*Pa*CO_2_) in the ranges of 10–15 kPa, and 4.0–6.5 kPa, respectively. The EBF rate was set to 1 L/min to prevent circuit coagulation until the start of the experiment^7,21^.

### Vital functions and haemodynamic monitoring

Vital functions and haemodynamic monitoring used in our study has been described previously^7,21^. Briefly, arterial pressure was measured using standard invasive methods with fluid-filled pressure transducers (Truwave, Edwards Lifesciences LLC, USA) through a pigtail catheter inserted into the aortic arch. A Swan-Ganz catheter was inserted into the pulmonary artery via the femoral vein. Electrocardiographic parameters, heart rate (HR), invasive blood pressures (aortic arch and central vein), pulse oximetry, capnometry, and invasive central venous SpO_2_ were continuously monitored in all animals (Monitor Life Scope TR, Nihon Kohden, Japan; and Vigilance II, Edwards Lifesciences, USA). Four-channel NIRS (i.e., near-infrared spectroscopy) oximetry (INVOS, Medtronic, Minneapolis, MN, USA) was used to monitor regional tissue perfusion (brain, front limbs, body, hind limb); the threshold for the detection of hypoperfusion was 40%^7,21^.

### Pressure–volume analysis

A pressure–volume (PV) conductance catheter (Scisense 7 Fr VSL Pigtail, Transonic, Ithaca, NY, USA) was inserted into the left ventricle from the left carotid artery through the aortic valve to monitor cardiac performance during induction and maintenance of CS as described elsewhere^7,22^. Correct positioning was assessed radiographically and by verifying optimal PV loop morphology. The catheter was connected to the PV unit (Scisense ADV 500, Transonic, USA) and operated in the admittance mode. The volume was calibrated according to baseline pulmonary thermodilution (Combo CCO catheter, Edwards Lifesciences, Irvine, CA, USA). PV values were recorded continuously during the experiment. At each EBF level, values from five loops were taken at end-expiration, averaged, and used for further analysis. The collected PV data included LV end-diastolic pressure (LVEDP), LV end-diastolic volume (LVEDV), LV end-systolic volume (LVESV), dP/dt_max_, and arterial elastance (Ea)^7,22^.

LV stroke volume (LVSV) was calculated as LVSV = LVEDV − LVESV; LV ejection fraction (LVEF) was calculated as LVEF = LVSV/LVEDV; cardiac output (CO) was calculated as CO = LVSV × HR; and cardiac index (CI) was calculated as CI = CO/body surface area^7,22^.

### Cardiogenic shock (CS) model

After initiation of ECMO, the animals were stabilized for 10 min. During this time period, oxygen/air flow and fluid infusion rate were repeatedly adjusted to reach and maintain target *P*aO_2_, *P*aCO_2_, and right atrial pressure. Mechanical ventilation was subsequently switched to the CMV mode (respiratory rate, 5 breaths/min; inspiratory volume, 100 mL; and fraction of inspired oxygen, 0.21), which precipitated severe hypoxemia in the blood entering the left chambers of the heart and, in turn, caused tissue hypoxia in all tissues perfused by LV ejections, including the coronary arteries. The resulting global myocardial hypoxia rapidly lowered cardiac contractility, LVEF, and arterial blood pressure. Concurrently, the lower body was perfused with fully oxygenated blood from ECMO entering the circulation via the femoral artery. During the hypoxic period, EBF was gradually increased to maintain a mean arterial pressure (MAP) > 60 mmHg, thereby ensuring adequate perfusion pressure. After approximately 1 h of myocardial hypoxia, the haemodynamic criteria for severe CS were fulfilled, including LVEF, which decreased to < 30%, and CO, which decreased to < 3.5 L/min. Thereafter, continuous perfusion of the heart with hypoxaemic blood maintained advanced myocardial dysfunction and severely compromised haemodynamic function. If cardiac performance decreased further with a risk for circulation collapse, the target level of CS severity was adjusted with a partial increase in ventilation^21^.

### AS

Severe AS was simulated by the insertion of a valvuloplasty balloon (True, BD Biosciences, Franklin Lakes, NJ, USA) across the aortic valve with subsequent stepwise inflation under fluoroscopic and ultrasound control to reach transaortic peak pressure gradient ˃ 40 mmHg. The valvuloplasty balloon was removed after the measurements.

### MR

Mechanical destruction of the mitral valve leaflets and/or chordae tendineae was performed under fluoroscopic and ultrasound guidance using endoscopic grasping forceps (Olympus, Tokyo, Japan) inserted via the femoral vein and trans-septal approach to the left atrium. Development of severe MR was confirmed using echocardiography.

### Experimental protocol

**Figure 1:**
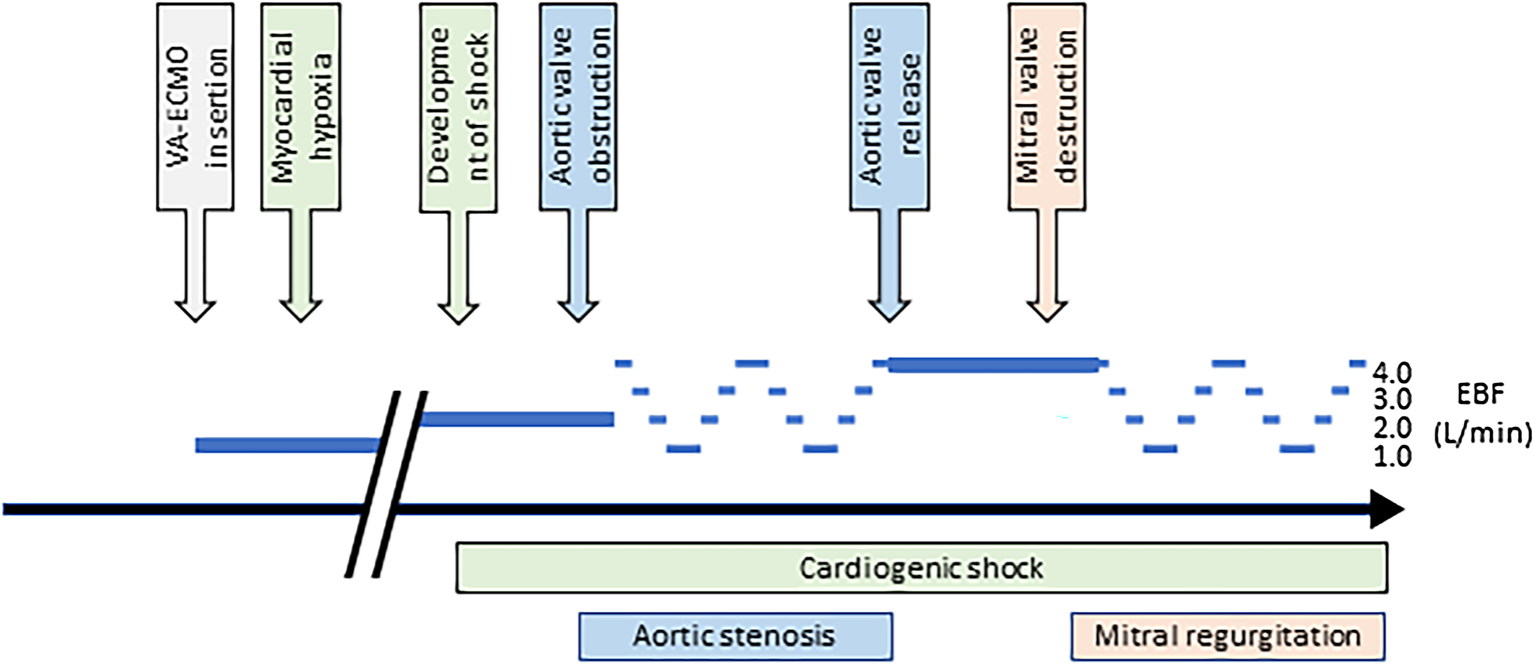
Schematic illustration of the experimental protocol. VA-ECMO, veno-arterial extracorporeal membrane oxygenation; EBF, extravascular blood flow.

Figure 1 outlines the experimental protocol that is similar to our previous studies^7,22^. After the placement of all catheters and the establishment of ECMO, the animals were permitted to stabilize for 10 min. CS was induced using global myocardial hypoxia, as described above. Following the occurrence of signs of tissue hypoperfusion (NIRS oximetry, < 40%) and an additional 10 min of stabilization, AS was simulated as described above, and the EBF rate was then set to 4 L/min and gradually decreased by 1 L/min every 5 min. Once an EBF rate of 1 L/min was achieved, it was maintained for 10 min. Subsequently, the EBF rate was gradually increased by 1 L/min every 5 min. At an EBF rate of 4 L/min, the animals were stabilized again for 10 min, and a second cycle of stepwise EBF decrease and increase was performed. After the measurements with AS were completed, the valvuloplasty balloon was removed from the aortic valve and MR was created (as described above) followed by 10 min stabilization. Subsequently, an additional two cycles of stepwise EBF decrease and increase were performed to record data for MR (Fig. 1). LV performance variables were analyzed at the end of each 5 min interval (four data sets per animal), and a mean value was calculated and used in further analysis.

### Statistical analysis

Data are expressed as median (interquartile range). The Friedman test was used to compare values at different levels of EBF; differences with P < 0.05 were considered to be statistically significant. All statistical analyses were performed using GraphPad Prism version 8.4 (GraphPad, San Diego, CA, USA).

### Ethics approval and consent to participate

This study was approved by the Charles University First Faculty of Medicine Institutional Animal Care and Use Committee, and was performed at the Animal Laboratory, Department of Physiology, First Faculty of Medicine, Charles University in Prague and Na Homolce Hospital, Prague, Czech Republic, in accordance with Act No 246/1992 Coll., for the protection of animals against cruelty. The investigation and protocol conformed to the Guide for the Care and Use of Laboratory Animals published by the United States National Institutes of Health (Publication No. 85-23, revised 1985). The study is reported in accordance with ARRIVE guidelines (https://arriveguidelines.org).

## Results

### Baseline characteristics

All animals survived and completed all protocol-defined procedures. Myocardial hypoxia resulted in a marked decline in MAP, CO, CI, LVSV and LVEF, while HR significantly increased (Table 1).

**Table 1:** Major hemodynamic variables at baseline and after the development of cardiogenic shock.

Variable	Baseline	Cardiogenic shock	P-value
MAP (mmHg)	90.2 (80.6–98.8)	47.6 (38.9–54.2)	< 0.01
CO (L/min)	6.1 (5.2–7.4)	2.7 (1.9–3.1)	< 0.01
CI (L/min/m^2^)	6.9 (5.8–8.3)	3.0 (2.1–3.48)	< 0.01
SV (mL)	72.2 (68.5–75.1)	22.2 (15.4–29.2)	< 0.01
HR (beats/min)	89.0 (79.1–104.9)	109.6 (98.5–124.2)	< 0.01
LVEF (%)	55.5 (53.3–59.0)	15.2 (10.5–19.3)	< 0.01

Data presented as median (interquartile range) unless otherwise indicated.

*CO* cardiac output, *HR* heart rate, *LVEF* left ventricular ejection fraction, *MAP* mean arterial pressure, *SV* stroke volume.

### AS

**Figure 2:**
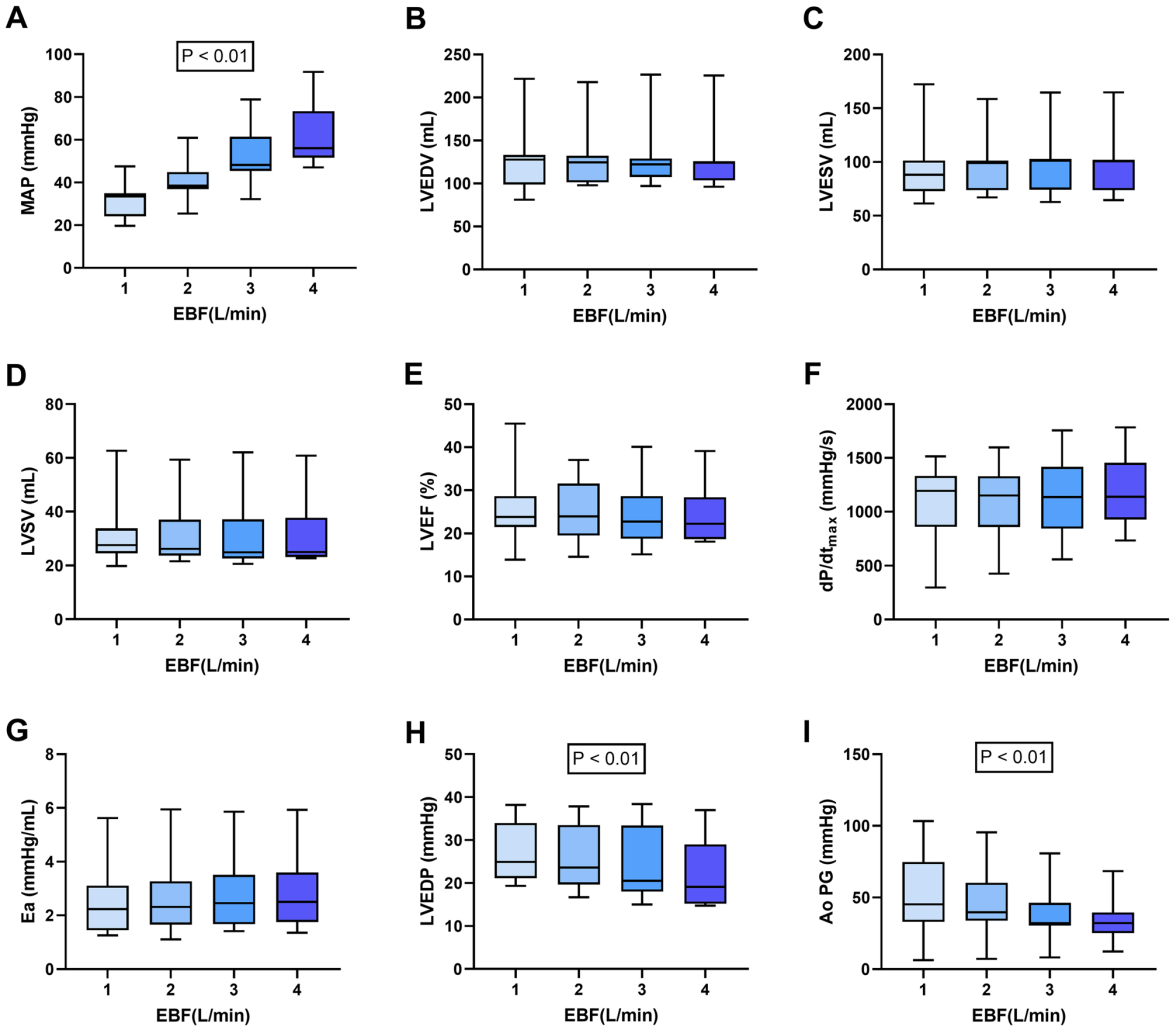
Comparison of the effect of different levels of venoarterial extracorporeal membrane oxygenation (VA-ECMO) blood flow (EBF) on hemodynamic and left ventricular performance parameters in a porcine model of cardiogenic shock with aortic stenosis. (**A**) MAP, mean arterial pressure; (**B**) LVEDV, left ventricle end-diastolic volume; (**C**) LVESV, left ventricle end-systolic volume; (**D**) LVSV, left ventricle stroke volume; (**E**) LVEF, left ventricle ejection fraction; (**F**) dP/dt_max_; (**G**) Ea, arterial elastance; (**H**) LVEDP, left ventricle end-diastolic pressure; (**I**) AoPG, peak aortic pressure gradient.

The stepwise increase in EBF was associated with a significant rise in MAP: 33.5 mmHg (24.2–34.9 mmHg) at EBF 1 L/min; 38.4 mmHg (36.9–44.9 mmHg) at EBF 2 L/min; 48.2 mmHg (45.4–61.3 mmHg) at EBF 3 L/min; and 56.0 mmHg (51.9–73.3 mmHg) at EBF 4 L/min (P ˂ 0.01) (Fig. 2A). LV volume variables (LVEDV, LVESV, LVSV), LVEF, dP/dt_max_ and Ea remained stable at different levels of EBF (Fig. 2B–G). LVEDP significantly decreased with increasing levels of EBF: 24.9 mmHg (21.2–40.0 mmHg) at EBF 1 L/min; 23.6 mmHg (19.7–33.5 mmHg) at EBF 2 L/min; 20.5 mmHg (18.0–33.4 mmHg) at EBF 3 L/min; and 19.1 mmHg (15.2–29.0 mmHg) at EBF 4 L/min (P ˂ 0.01) (Fig. 2H). The median aortic valve pressure gradient achieved significantly dropped with increasing levels of EBF, from 45.2 mmHg (33.0–74.8 mmHg) at EBF 1 L/min to 32.1 mmHg (25.1–39.6 mmHg) at EBF 4 L/min (P ˂ 0.01) (Fig. 2I).

### MR

**Figure 3:**
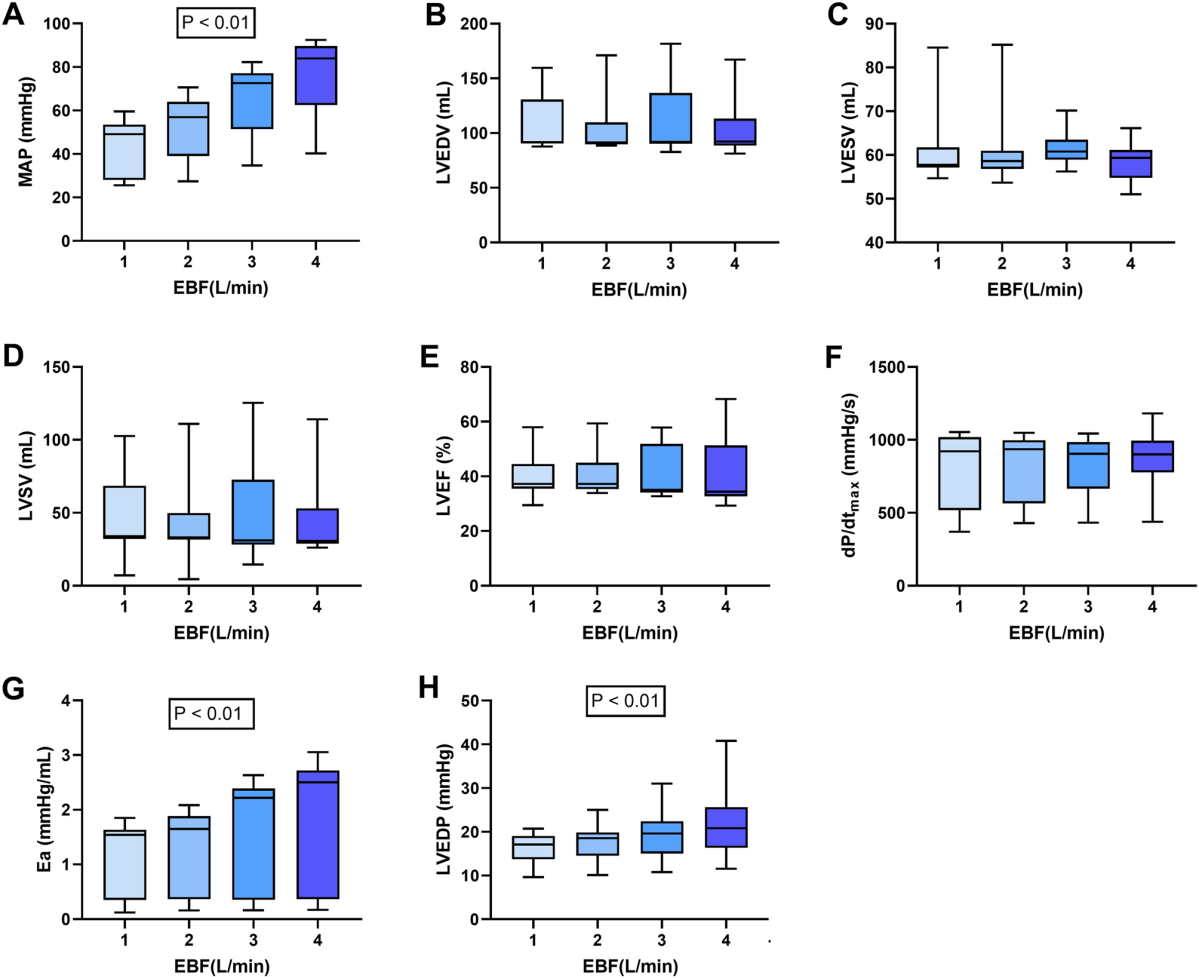
Comparison of the effect of different levels of venoarterial extracorporeal membrane oxygenation (VA-ECMO) blood flow (EBF) on hemodynamic and left ventricular performance parameters in a porcine model of cardiogenic shock with mitral regurgitation. (**A**) MAP, mean arterial pressure; (**B**) LVEDV, left ventricle end-diastolic volume; (**C**) LVESV, left ventricle end-systolic volume; (**D**) LVSV, left ventricle stroke volume; (**E**) LVEF, left ventricle ejection fraction; (**F**) dP/dtmax; (**G**) Ea, arterial elastance; (**H**) LVEDP, left ventricle end-diastolic pressure.

A gradual increase in EBF resulted in a significant elevation in MAP: 49.0 mmHg (28.0–53.4 mmHg) at EBF 1 L/min; 56.9 mmHg (39.0–64.0 mmHg) at EBF 2 L/min; 72.5 mmHg (51.4–77.1 mmHg) at EBF 3 L/min; and 84.0 mmHg (62.4–89.6 mmHg) at EBF 4 L/min (P ˂ 0.01) (Fig. 3A). LV volume variables (LVEDV, LVESV, LVSV), LVEF and dP/dt_max_ remained stable at different EBF rates (Fig. 3B–F). Median Ea increased from 1.5 mmHg/mL (0.4–1.6 mmHg/mL) at EBF 1 L/min to 2.5 mmHg/mL (0.4–2.7 mmHg/mL) at EBF 4 L/min (P ˂ 0.01) (Fig. 3G). Median LVEDP significantly rose with increasing EBF: 17.1 mmHg (13.7–19.1 mmHg) at EBF 1 L/min; 18.5 mmHg (14.5–19.9 mmHg) at EBF 2 L/min; 19.6 mmHg (15.0–22.4 mmHg) at EBF 3 L/min; and 20.8 mmHg (16.3–25.6 mmHg) at EBF 4 L/min (P ˂ 0.01) (Fig. 3H).

## Discussion

To the best of our knowledge, the present study was the first to directly address the effect of VA-ECMO on LV function in CS with AS or MR. There were two major findings. First, in CS with AS, the effect of increased VA-ECMO blood flow on LV performance parameters was either neutral (LVEDV, LVESV, LVSV, LVEF) or even beneficial (LVEDP decrease). Second, in CS with MR, increased EBF, MAP and Ea did not affect LV volume variables (i.e., LVEDV, LVESV, LVSV, LVEF), but increased LVEDP. Therefore, AS prevented LV overload and partial protection was observed also with MR, at the cost of higher LVEDP.

There are numerous factors that can influence or modulate the effect of VA-ECMO on LV performance including severity of LV dysfunction, EBF rate, use of vasopressors, inotropes, mechanical ventilation setting, presence of right heart failure, and the competence of the heart valves. Valvular heart disease is often present in patients with CS, either with causal relationship or as a concomitant and modulating disease. Severe AS can be a cause of CS, especially if decompensated with LV systolic dysfunction, or contribute to shock in case of acute myocardial injury of other etiology (e.g., myocardial infarction or myocarditis). Additionally, acute MR can clinically manifest as CS, and severe MR is often present as concomitant disease as a result of LV dilatation in advanced heart failure^16–18^.

Increased VA-ECMO blood flow in CS with severe LV dysfunction and competent valves predominantly increases LVESV and reduces LVSV and LVEF; this phenomenon is largely explained by increased LV afterload at higher EBF rates^7,14,22^. However, if severe AS is present, LV afterload is determined almost entirely by obstruction of the aortic valve and increased VA-ECMO blood flow does not translate into an elevation in LV afterload as demonstrated in our study where Ea was not significantly changed. This could also be a reason why we did not observe any changes in LV volumes with increasing EBF during the simulation of AS. Moreover, decreased preload caused by increased blood drainage from the right atrium at higher EBF can be responsible for reduced LVEDP in our study. Therefore, our data indicate that, in CS with severe AS, VA-ECMO restores systemic circulation and, at the same time, unloades the left ventricle, prevents LV overload, and reduces the risk for pulmonary oedema. In clinical practice, VA-ECMO is frequently used as circulatory support in CS with AS^23–27^, and our data support this approach from the haemodynamic perspective.

The situation is different in CS with severe MR. Increased EBF is associated with higher Ea and an incompetent mitral valve enables translation of the effect of increased LV afterload to the left atrium. We did not record regurgitation volumes; however, we speculate that increased LV afterload at higher EBF resulted in increased regurgitation volume, which would fully explain our observation of unchanged LVESV and LVSV together with increased LVEDP. Importantly, higher LVEDP is associated with an increased risk for subsequent pulmonary oedema. Thus, preservation of LV function (LV volumes and LVEF) in CS with MR does not necessarily lead to stable haemodynamic conditions but more likely increased congestion. This effect could be even greater in predominantly LV failure where preserved RV would contribute to increased LV preload, hence increased LVEPD. Additionally, atrial septostomy (transseptal puncture), that we had to performe when introducing MR model, may have aleviated pulmonary congestion to some extent by venting left atrium^28^. Furthermore, because LVSV was calculated from the PV loop and includes both transaortic flow volume and mitral regurgitation volume, stable LVSV does not reflect reduced transaortic flow and thus lower native CO at higher EBF. Our results, therefore, support the recommendation to not use VA-ECMO alone in MR but rather in combination with other devices such as the Impella system^18^.

Our study had several limitations. We used a model of CS caused by global hypoxia that affects not only the left but also the right ventricle, causing severe biventricular systolic dysfunction. Therefore, our model differs from other large animal models of acute heart failure, which are primarily based on the development of myocardial infarction by coronary artery occlusion; however, it reflects frequent clinical scenarios. On the other hand, acute MR or AS usually do not result in immediate right ventricular systolic dysfunction and therefore, hemodynamic consequences of cardiogenic shock caused by acute myocardial infarction may be different then in shock caused by hypoxia affecting both ventricles. Furthermore, LV ejections in our model supply with the hypoxemic blood not only coronary arteries but also carotid arteries at least during the initial phases of the hypoxic period. Hypoxic brain damage could therefore be anticipated. We cannot exclude that cerebral hypoxia may have influenced some of the mechanisms of central regulation of blood circulation. Moreover, we focused on the acute effects of VA-ECMO on haemodynamic and LV performance variables. We speculate that, especially in severe MR, long-term use of VA-ECMO may result in an increase in LV volumes and LV dilatation. A specific limitation of our study is also the non-administration of vasopressors even at very low blood pressure, which is not in accordance with clinical practice. Another important limitation of our study is the simulation of AS by balloon obstruction of aortic valve and MR by mechanical destruction of the mitral valve which does not reflect the processes that happen in real-life valvular heart disease patients with varying degrees of aortic valve opening area, and where the mitral valve is usually not destructed, but tethered and dilated (or showing prolapsing or flail segments), leading to significant variations in the degree of MR. Both AS and MR in our study were also simulated sequentially in each individual animal; however, since the aortic valve obstruction was completely reversible and preceded the development of MR, it is unlikely that the observations in MR were significantly influenced by previous AS simulation. Our study also includes only eight experimental animals; however, changes in MAP and LV performance variables were uniformly expressed across all individual subjects. Finally, our experimental study was conducted in young and otherwise healthy animals. Therefore, caution is advised in translating our results to clinical scenarios involving patients with advanced heart failure, LV remodelling, and comorbidities.

## Conclusion

Results of the present study indicate that the presence of valvular heart disease may alleviate the negative effect of VA-ECMO on LV performance. In CS with severe AS, VA-ECMO restored systemic circulation together with LV unloading and prevention of pulmonary oedema. In acute CS with severe MR, the restoration of systemic blood flow was associated with acutely preserved LV volumes and LVEF at the cost of increased LVEDP and, thus, higher risk for pulmonary oedema.

## Data Availability

The datasets used and/or analysed during the current study are available from the corresponding author on reasonable request.

## Abbreviations

AS: Aortic stenosis
CO: Cardiac output
CO_2_: Carbon dioxide
CS: Cardiogenic shock
EBF: Extracorporeal blood flow
IV: Intravenous
LV: Left ventricle
LVEDP: Left ventricular end-diastolic pressure
LVEDV: Left ventricular end-diastolic volume
LVEF: Left ventricular ejection fraction
LVESV: Left ventricular end-systolic volume
LVSV: Left ventricular stroke volume
MAP: Mean arterial pressure
MR: Mitral regurgitation
NIRS: Near-infrared spectroscopy
PaCO_2_: Partial pressure of CO_2_
PaO_2_: Partial pressure of oxygen
PV: Pressure-volume
SpO_2_: Oxygen saturation
VA-ECMO: Venoarterial extracorporeal membrane oxygenation
